# Supplementary material for: The causal role of circulating inflammatory markers in osteoporosis: a bidirectional Mendelian randomized study
Source: Front Immunol. 2024 Jul 18;15:1412298. doi: 10.3389/fimmu.2024.1412298 (PMC11291241; doi:10.3389/fimmu.2024.1412298)
Supplement: Supplementary file 1 [file DataSheet_1.zip › supplementary material/OSTEOPOROSIS-CIM/GCST90274815.tsv.gz_leave_one_out.pdf]

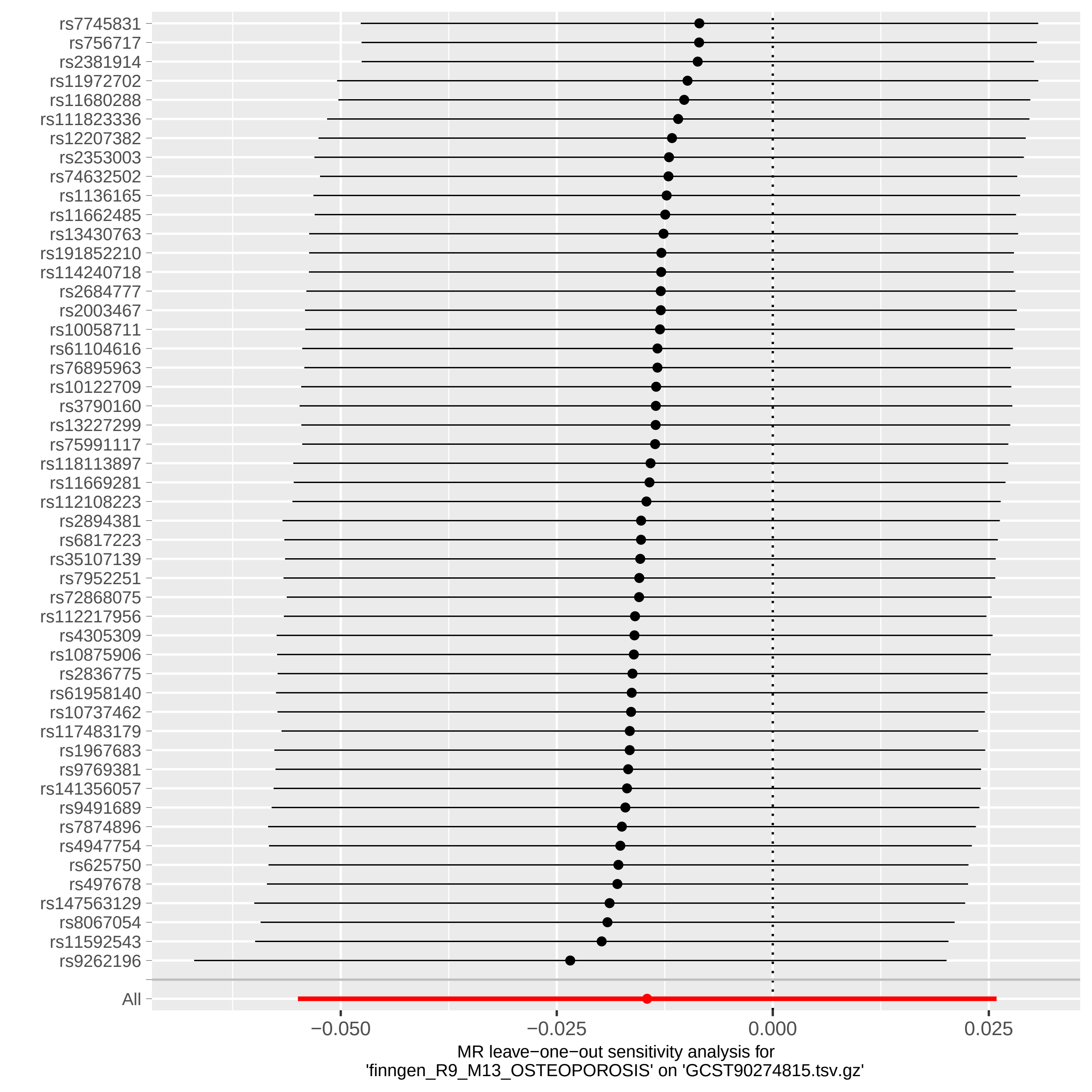
rs7745831
rs756717
rs2381914
rs11972702
rs11680288
rs111823336
rs12207382
rs2353003
rs74632502
rs1136165
rs11662485
rs13430763
rs191852210
rs114240718
rs2684777
rs2003467
rs10058711
rs61104616
rs76895963
rs10122709
rs3790160
rs13227299
rs75991117
rs118113897
rs11669281
rs112108223
rs2894381
rs6817223
rs35107139
rs7952251
rs72868075
rs112217956
rs4305309
rs10875906
rs2836775
rs61958140
rs10737462
rs117483179
rs1967683
rs9769381
rs141356057
rs9491689
rs7874896
rs4947754
rs625750
rs497678
rs147563129
rs8067054
rs11592543
rs9262196
All
−0.050
−0.025
0.000
0.025
MR leave−one−out sensitivity analysis for
'finngen_R9_M13_OSTEOPOROSIS' on 'GCST90274815.tsv.gz'
